# Impact of changing PI-RADS cutoff on prostate cancer detection by MRI cognitive fusion biopsy in biopsy-naïve patients

**DOI:** 10.1186/s43046-023-00165-4

**Published:** 2023-03-06

**Authors:** Hesham Abdel-Azim El-Helaly, Asem Abdel-Aziz Mahmoud, Ahmed Mohamed Magdy, Abdelwahab Hasehem, Hamdy Mohamed Ibrahim, Khaled Moheyelden Mohamed, Mohamed Hamdy Ismail

**Affiliations:** 1 Urology Department, Fayoum University, Fayoum, Egypt; 2 Radiology Department, Fayoum University, Fayoum, Egypt; 3 Urology Department, Urology and Nephrology Center, Mansoura, Egypt; 4 Urology Department, 30th June Urology and Nephrology Center, Ismailia, Egypt; 5 Urology Department, Shebin Elkom Teaching Hospital, Shebin Elkom, Egypt

**Keywords:** Prostate cancer, Biopsy, TRUS, Diagnosis, Oncology

## Abstract

**Background:**

Multi-parametric magnetic resonance imaging may improve the detection of prostate cancer. The aim of this work is to compare between PI-RADS 3–5 and PI-RADS 4–5 as a threshold for targeted prostatic biopsy.

**Methods:**

This is a prospective clinical study that included 40 biopsy-naïve patients referred for prostate biopsy. Patients underwent prebiopsy multi-parametric (mp-MRI), followed by 12-core transrectal ultrasound-guided systematic biopsy and cognitive MRI/TRUS fusion targeted biopsy from each detected lesion. The primary endpoint was to assess the diagnostic accuracy of the PI-RAD 3–4 versus PI-RADS 4–5 lesion by mpMRI for prostate cancer detection in biopsy-naive men.

**Results:**

The overall prostate cancer detection rate and the clinically significant cancer detection rate were 42.5% and 35%, respectively. Targeted biopsies from PI-RADS 3–5 lesions showed a sensitivity of 100%, specificity of 44%, positive predictive value of 51.7%, and negative predictive value of 100%. Restricting targeted biopsies to PI-RADS 4–5 lesions resulted in a decrease in sensitivity and negative predictive value to 73.3% and 86.2%, respectively, while specificity and positive predictive value were increased to 100% for both parameters which was statistically significant (*P* value < 0.0001 and *P* value = 0.004, respectively).

**Conclusions:**

Limiting the TBs to PI-RADS 4–5 lesions improves the performance of mp-MRI in the detection of prostate cancer especially aggressive tumors.

## Background

At the present time, systematic 12-core transrectal ultrasound (TRUS)-guided biopsy is the standard of care for the diagnosis of prostate cancer (PCa). Accordingly, prostatic carcinoma is the only tumor that is dependent on its diagnosis on random instead of targeted sampling. Despite that, the situation is unsatisfactory because of defects in PCa detection and targeted sampling under the guidance of new imaging methods looks appealing solution to this problem [1].

Recently, multi-parametric magnetic resonance imaging (mp-MRI) has been introduced as a hopeful tool in the quest to avoid over and underdiagnosis of PCa cases. The most modest among the MRI-guided methods is the cognitive fusion which depends on mental combination of MRI targets to the real-time TRUS study to guide the sampling process. The European Association of Urology (EAU) has 2 propositions for using mp-MRI-guided biopsy in PCa diagnostic pathway. The first is to promote diagnosis of clinically significant prostate cancer (csPCa) by combined (targeted plus systematic) biopsy in cases with MRI containing lesions and only systematic biopsies if the MRI had no lesions. The second way is to use MRI as a sorting method to select candidates for biopsy where cases who have lesions in their MRI perform targeted biopsy only and cases who do not have lesions would avoid prostatic biopsy [2–4].

With the development of reporting system, PI-RADS version 2, there is hope that targeting biopsy for PI-RADS 3–5 lesion can increase the csPCa detection rate and decrease clinically insignificant prostate cancer (ciPCa) detection rate in comparison to the systematic TRUS biopsy [5].

The aim of this study is to evaluate the diagnostic accuracy of the mpMRI-based approach to the standard approach in biopsy-naive men.

## Methods

This was a prospective study carried out at a Referral Urology Department of University Hospital, during the period from April 2018 to December 2019.

The inclusion criteria were (1) age between 40 and 70 years, (2) suspicious digital rectal examination (DRE), (3) elevated prostate-specific antigen (PSA) level > 4 ng/ml confirmed not to be due to urinary tract infection or recent prostatic manipulation, and (4) informed written consent. The exclusion criteria were (1) previous prostatic biopsy, (2) known prostatic cancer, (3) follow-up after prostatic cancer treatment, (4) contraindicated for MRI (e.g., with metallic implants or cardiac pace-maker), and (5) contraindicated for prostatic biopsy (e.g., coagulopathy, severe immune-suppression, acute prostatitis, and sever anal stenosis).

The study protocol was approved by the Faculty of Medicine Ethics Council and conducted in accordance with the principles of the Declaration of Helsinki. All patients provided written informed consent prior to enrollment.

Patients underwent systematic biopsies (SBs) plus cognitively targeted transrectal biopsies (TBs) from PI-RADS 3, 4, and 5 lesions with 2 cores from each lesion, preserved separately. In case of PI-RADS 1 and 2, only systematic biopsies were taken. Targeted biopsies were taken by one urologist followed in the same session by systematic biopsies performed by a separate urologist blinded to the MRI result.

Patients were examined in a supine position by using (Vantage Titan 1.5 T, Toshiba Medical Systems, Tochigi, Japan) equipment and pelvic phased-array surface coil. The mp-MRI sequences used for patients were T1-weighted imaging (T1WI), axial T2-weighted imaging (T2WI), T2 fat saturation (T2 FAT SAT), coronal T2WI, sagittal T2WI, axial diffusion-weighted imaging (DWI) with apparent diffusion coefficient (ADC) map, and axial dynamic contrast-enhanced (DCE) MRI. The volume of the prostate gland was measured using the prolate ellipse formula: volume = height × width × length × 0.52. Assessment, reporting, and mapping of lesions were done using the Prostate Imaging Archiving and Reporting Data System (PI-RADS™ v2) [6] by a dedicated senior radiologist (Magdy A.M.) with an 8-year experience in prostate MRI reading.

Transrectal prostatic imaging was carried out using a 6-MHz, 150° end-firing probe (PVG-630 V) mounted on ultrasound device (Toshiba Famio-5 SSA-510A; Toshiba Medical Systems Corporation, Tochigi, Japan). The prostate volume was calculated using the same formula used in MRI. The gland, including the seminal vesicles (SV) and ejaculatory ducts (ED), was then scanned systemically in axial and sagittal planes. Abnormalities were viewed in both planes for confident analysis.

In this study, csPCa was defined as biopsy Gleason score (GS) ≥ 7 (3 + 4), more than 2 cores involved or any cancer core length (CCL) longer than 5 mm [7]. Intermediate and high risk PCa was defined according to modified criteria of International Society of UroPathology (ISUP 2014) as tumors ≥ ISUP grade 2 [8].

### Statistical analysis

The collected data were organized, tabulated, and statistically analyzed using SPSS software (Statistical Package for the Social Sciences) version 22 (SPSS Inc., USA). Quantitative data were presented as mean ± SD, or median and Inter quartile range (IQR). Student’s *t* test and one-way ANOVA test were used as test of significance to compare between two and three groups, respectively. Qualitative data were presented as numbers and percentages. The chi-square (*χ*^2^) or Fischer exact test was used as a test of significance. Among the MRI group, sensitivity, specificity, positive predictive value (PPV), and negative predictive value (NPV) were calculated to assess the validity of PI-RADS score in detecting PCa. Also, Cohen’s kappa (*κ*) was performed to determine the correspondence between targeted biopsy (TB) and systematic biopsy (SB) regarding histopathological diagnosis. A probability value (*P* value) ≤ 0.05 was considered to be a statistically significant.

## Results

The mean age of the participants was 65 years ± 6.6 (range 49–76), the median PSA was 12.5 ng/mL (interquartile range [IQR] 7.8–16.4), the median prostatic volume was 82.3 cc (IQR 58.1–116), and 8 patients had suspicious DRE.

The detection rates of PCa, csPCa, and intermediate/high-risk PCa in the study group were 17/40 (42.5%), 14/40 (35%), and 9/40 (22.5%), respectively. In this study, 544 cores were retrieved study population. The cancer detection rate among them was 111/544 (20.4%).

Positive MRI was defined as containing lesion scored ≥ PI-RADS 3. There were 29 cases with positive MRIs containing 32 lesions. The detection rate of PCa and csPCa among MRI positive vs MRI negative cases was 15/40 vs 2/40 (*P* = 0.045) and 13/40 vs 1/40 (*P* = 0.034), respectively (Table 1).

**Table 1:** Comparison histopathologic result of MRI + ve and MRI − ve

MRI	MRI + ve, target	MRI –ve, no target	*p* value
Group size (cases)	29	11	–
PCa detection rate	15/29 (51.7%)	2/11 (18.2%)	0.045^**#**^
Detection rate of csPCa	13/29 (44.8%)	1/11 (9%)	0.034^**#**^
Detection rate of intermediate/high-risk PCa	8/29 (27.6%)	1/11 (9%)	0.211^#^

Data were presented as numbers and percentages

^#^Chi-square test/Fisher’s exact test

Among 32 targeted lesions in study patients, 19, 8, and 5 lesions were scored as PI-RADS 3, 4, and 5, respectively. The detection rate of PCa and intermediate/high-risk PCa among 3, 4, and 5 scores were 4/19, 8/8, and 5/5, respectively (*P* = 0.001) and 0/19, 6/8, and 4/5, respectively (*P* < 0.001).

TBs and SBs showed an agreement in 35/40 cases regarding histopathological diagnosis which was statistically significant (Cohen’s kappa (*κ*) = 0.779 and *P* < 0.0001). RBs detected 2 PCa cases missed by TBs. TBs detected 2 PCa cases missed by RBs and upgraded 1 case scored as ISUP 2 by RBs to ISUP 3.

Setting PI-RADS 3–5 as a threshold to take TBs resulted in a decrease in number of men requiring biopsy and number of examined cores by 27.5% and 86.7%, respectively. Regarding cancer detection, there was no change in the overall PCa detection, decrease in PCa ≥ ISUP 2 by 11.1% and increase in PCa = ISUP 1 by 16.7%, (Table 2). Also, TBs had sensitivity, specificity, PPV, and NPV by 100%, 44%, 51.7%, and 100%, respectively (Table 3).

**Table 2:** Comparison of biopsy outcomes with two different approaches: (a) SBs vs TBs from lesions scored PI-RADS 3**–**5 and (b) SBs vs TBs from lesions scored PI-RADS 4–5

PI-RADS 3–5	SBs	TBs	Difference %
Pt. no	40	29	− 27.5%
Biopsy cores no	480	64	− 86.7%
PCa detection rate	15	15	0%
I/H risk PCa detection rate	9	8	− 11.1%
Low-risk PCa detection rate	6	7	16.7%
PI-RADS 4–5	SBs	TBs	Difference %
Pt. no	40	11	− 72.5%
Biopsy cores no	480	26	− 94.6%
PCa detection rate	15	11	− 26.7%
I/H risk detection rate	9	8	− 11.1%
Low-risk detection rate	6	3	− 50.0%

**Table 3:** Statistical performance of TBs of PI-RADS 4–5 versus PI-RADS 3–5 lesions in the MRI group

	PI-RADS 3–5	PI-RADS 4–5	*P* value
	% (95% CI)	% (95% CI)	
Sensitivity	100.0% (78.2–100)	73.3% (44.9–92.2)	0.100^#^
Specificity	44.0% (24.4–65.1)	100.0% (86.0–100)	< 0.0001^**#**^
PPV	51.7% (32.5–70.6)	100.0% (71.5–100)	0.004^**#**^
NPV	100.0% (71.5–100)	86.2% (68.3–96.1)	0.560^#^

Data were presented as percentages and 95% confidence interval

^#^Fisher’s exact test

Changing the threshold of taking TBs to PI-RADS 4–5 would result in a decrease in the number of men requiring biopsy and the number of examined cores by 72.5% and 94.6% respectively. Regarding cancer detection, there was a decrease in the overall PCa detection by 26.7% which resulted because of decrease in PCa = ISUP 1 detection by 50% with no change regarding PCa ≥ ISUP 2. Also, specificity and PPV have significantly increased to 100% while sensitivity and NPV have decreased to 73.3% and 86.2%, respectively (Table 3).

## Discussion

Currently, targeting a predefined lesion seems logical to overcome the problem of overdiagnosis and overtreatment encountered in PCa. Cognitive targeting using prebiopsy mp-MRI is the simplest way in this regard. Cognitive fusion, though performed in many institutes, is performer dependent and subjective compared with robot-assisted MR fusion biopsy. The evidence about the impact of MRI in repeated biopsy cases is undeniable however in biopsy-naïve cases, it is controversial [9].

One of the methods suggested to use mp-MRI in the first biopsy setting is to be used as a triage test where cases with negative MRI can avoid biopsy. For this to be possible, mp-MRI should have a high NPV. In our study, the detection rates of PCa and csPCa among MRI-negative cases were 18% and 9%, respectively, which were significantly lower than MRI-positive cases (*P* = 0.045 and 0.034, respectively). The MRI showed a NPV for PCa of 100% when the cutoff of positive MRI was PI-RADS 3–5 and 86.2% for a cutoff PI-RADS 4–5.

A meta-analysis assessing 48 reports (involving 9613 men) found that the median NPV of mp-MRI regarding overall PCa was 82.4% and that regarding csPCa was 88.1% [10]. A more recent meta-analysis, assessing 42 studies including 7321 patients, revealed a NPV of mp-MRI for csPCa (ISUP ≥ 2) by 90.8% when the cutoff was PI-RADS 3–5 and 86.8% when the cutoff was PI-RADS 4–5 [11].

Equivocal lesions scored as PI-RADS 3 represent a special situation as there are no guidelines whether to take a biopsy or to follow up in biopsy-naïve cases. In the current study, the PI-RADS 3 lesions comprised 43% of the MRI group. The PCa detection rate among them was 21% which was significantly lower than that for PI-RADS 4–5 lesions (*P* = 0.001) and all of the diagnosed tumors were ISUP 1 which was also statistically significant (*P* < 0.001). A meta-analysis of 28 studies involving 1759 PIRADS 3 patients reported that PI-RADS 3 patients represented about 17.3% among studies. The detection rate of PCa was 44% (7.1–55.8%) while that of csPCa was 21.2% (3.4–46.5%) in biopsy-naïve patients [12].

Assessment of the performance of PI-RADS v2 and defining the optimal threshold score for taking a biopsy in cases with positive MRI is an important area of controversy. In our study, setting PI-RADS 4–5 as the definition of positive MRI and the threshold for targeted biopsy increased the specificity and PPV, yet decreased the sensitivity and NPV. Increase in specificity and PPV was statistically significant (*P* < 0.0001 and *P* = 0.004, respectively) while decrease in sensitivity and NPV were insignificant (*P* = 0.100 and *P* = 0.560). In a meta-analysis of 21 reports involving 3857 men, choosing PI-RADS 4–5 as a definition to positive MRI achieved sensitivity and specificity of 89% and 74% while changing the threshold to PI-RADS 3–5 improved sensitivity to 95%, but decreased specificity to 47%. The authors proposed utilizing PI-RADS score 4–5 as a threshold to carry out a prostatic biopsy in the situation of biopsy-naïve cases while score 3–5 is to be used in the setting of repeated biopsy after previously negative sampling to avoid missing any tumor [13].

One of the most debated questions is whether, in the presence of a positive mpMRI, TBs alone might be enough. In our study, combined strategy was effective in 12.5% of the cases that would have been missed or misdiagnosed. Supporters of the combined approach argue that there is an additional diagnostic yield of csPCa detection ranging from 5 to 20% [14]. In addition, obtaining histological information about prostate areas that are not suspicious on mpMRI is important as it can influence the margins and nerve-sparing approach in radical surgery [15].

Proponents of mpMRI-TBs alone strategy argue that the proportion of csPCa missed is low, and the RBs detect roughly double the number of ciPCa as mpMRI-TBs [16]. Moreover, mpMRI-TB is superior at detecting anterior and apical tumors [17]. Other advantages of the mpMRI-TBs alone approach include the reduction in core number, operative time, pathologist time, and patient-reported complications [9].

The main strength of this study was the involvement of two urologists in prostatic biopsy. The one involved in systematic biopsies was blinded to MRI results. TBs were performed before random biopsies to prevent the negative impact of biopsy-induced bleeding artifacts and gland swelling.

On the other hand, our study has some limitations. We lacked the correlation with a reference standard like specimen pathology or template prostate mapping. The problem is that radical prostatectomy specimens are highly selected since men must test positive for cancer on TRUS biopsy and choose to have surgery. Meanwhile, we do not have the expertise or the equipment to do transperineal mapping biopsy. In addition, comparisons between the subgroups might have been less reliable owing to the overoptimistic power calculation leading to an underpowered study and a small sample size that was not calculated.

## Conclusions

Improving the performance of mp-MRI by TBs to PI-RADS 4–5 lesions could detect aggressive tumors and might decrease the rate of over-diagnosis of clinically insignificant cancers.

## Data Availability

All data and materials are available if requested.

## Abbreviations

ADC: Apparent diffusion coefficient
AUC: Area under the curve
CCI: Cancer core invasion
CCL: Cancer core length
ciPCa: Clinically insignificant prostate cancer
csPCa: Clinically significant prostate cancer
DCE: Dynamic contrast enhancement
DRE: Digital rectal examination
DWI: Diffusion-weighted imaging
EAU: European Association of Urology
ED: Ejaculatory ducts
FAT SAT: Fat saturation
GS: Gleason score
ICC: Interclass correlation coefficient
IQR: Interquartile range
ISUP: International Society of UroPathology
mp-MRI: Multi-parametric magnetic resonance imaging
MRI: Magnetic resonance imaging
NPV: Negative predictive value
PCa: Prostate cancer
PI-RADS v2: Prostate Imaging, Reporting and Data System Version 2
PPV: Positive predictive value
PSA: Prostate-specific antigen
RB: Random biopsy
SBx: Systematic biopsy
SD: Standard deviation
SPSS: Statistical Package for the Social Sciences
SV: Seminal vesicles
T1W: T1-weighted image
T2W: T2-weighted image
TB: Targeted biopsy
TRUS: Transrectal ultrasound
